# The Medical Digest
*"The Medical Digest, or Busy Practitioners' Vade Mecum," being a means of readily acquiring information upon the principal contributions to medical science during the last fifty years. By Richard Neale, M.D. London, &c. Third edition. (London: Ledger, Smith, and Co., St. Mary Axe. 1891.)


**Published:** 1891-11-14

**Authors:** 


### Turning Over New Leaves.

### The Medical Digest.*

Mr. Gladstone, in one of his most recent non-political speeches, quotes that most learned ecclesiastic Dr. Döllinger's remark on Dr. William Smith's "Dictionary of the Church," that there was nothing like it in Germany. If we apply that quotation to the above work and say that there is nothing like it in the whole world of medical literature, we are well within the limits of truth. The book is the result of long years of patient work, which ought to have been done by some public state office, but which we owe to the determined perseverance of a member of that backbone of the profession, the general practitioner. Dr. Neale, by setting apart from the day's work a portion of time for this purpose, has succeeded in producing a volume which is not only useful but absolutely necessary to every man engaged in the busy routine of his profession.

It has been stated that the "Medical Digest" is an index merely. This is a great mistake. It is not only an index of, but an absolute reference to the treatment of disease which is stored in our chief medical journals for the last fifty years. When we consider that in that time the greatest conceivable changes have taken place in our ideas of disease and our methods of treatment, it will be seen at once how large an obligation the profession is under to Dr. Neale. So great now is the pressure of work that a busy medical man can hardly find time to get through his journals in the week, much less remember all the practical points that they contain; but the "Digest" does all this for him. Given an anxious case which does not get on, after all that the medical man himself knows has been done, he begins to think of aid from other brains than his, but before doing so he gives a glance at the Digest. Here he finds reference after reference to methods of treatment, and this not a mere index, but shrewdly compressed into a line, the pith of a whole dissertation. So our hard-pressed friend takes heart, and tries some of the many methods that lie open to him, and often, doubtless, with success and pleasure to himself and to his patient.

Again, a man is getting up a paper to be read before a learned society, or to be published in one of the great medical journals; that most tedious of all work—the bibliography of the subject—has to be gone through ;here the "Digest" steps in, and goes a long way towards helping him.

* "The Medical Digest, or Busy Practitioners' Vade Mecum," being a means of readily acquiring information upon the principal contributions to medical science during the last fifty years. By Richard Neale, M.D. London, &c. Third edition. (London: Ledger, Smith, and Co., St. Mary Axe. 1891.)

On looking through the book, it will be seen how vast the number of subjects is that it goes into. Not only purely medical and surgical, but everything of scientific interest which in the slightest way touches on the healing art.

One word as to the right way of using the book. The subjects are arranged under numbered headings, with sub-divisions. To these, as a whole, is a most copious index. This index has nothing to do with the paging of the book, which is only retained for the use of the printer and binder. Let us suppose we want some help with regard to a hip case. Well we turn up the index and read "Hip, 1,747—4," that is, section 1,747, paragraph 4. Suppose it was with regard to resection of that joint, the index gives us "Hip, resection 1753—4," on turning up that section we find no less than thirty-nine references, and so on with regard to every imaginable emergency that our varied profession may land us in. Not only are the references given, but the methods of treatment, with the names of their discoverers are mentioned, so that if time does not suffice to go directly to the volume from which the reference is taken, we still have the salient points before us, and are enabled to do what is required. In fact, to quote from a review of a previous edition, "the 'Digest' at once affords ample information without the necessity of further reference. It is impossible to say where the indebtedness of those who have the good fortune to possess the 'Digest' will end, for no day probably passes in which it does not serve in some way to assist."

In conclusion, it may well be asked what has been done in the way of honouring the author of this invaluable work? The answer, as far as regards the outside world, is—nothing. But from the body of the profession of which he is such a pillar comes a chorus of grateful thanks. Of him the tired family doctor, hard pressed with his ceaseless round of visiting the sick, thinks kindly as he turns up that reference which leads to relief of pain or suffering, and helps to remove that weight of anxiety which always presses more or less on him.

Of him the young and struggling consultant, who is toiling over that work which is to lead to the greater recognition of his talents from the profession at large, thinks gratefully as column after column of notes on his subject lies before him ready to be used at once, and rendering unnecessary that almost waste of time in collecting a bibliography. Of him, the older man, be he consultant or general practitioner, has a genial sense of service rendered as he glances over the list of methods familiar to him as a young physician, and notes the changes that have come over the profession in his time. So, in taking a farewell glance at our author, can it be said of him that he has built for himself "*monumentum ære perennius.*"

